# Clinical Outcomes of Upper Eyelid Blepharoplasty

**DOI:** 10.12669/pjms.41.5.10950

**Published:** 2025-05

**Authors:** Murtaza Sameen, Rebecca

**Affiliations:** 1 Murtaza Sameen Assistant Professor, Al Shifa Trust Eye Hospital, Rawalpindi, Pakistan; 2 Rebecca Senior Registrar, Al Shifa Trust Eye Hospital, Rawalpindi, Pakistan

**Keywords:** Aesthetic Surgery, Blepharoplasty, Dermatochalasis, Upper eyelid, Xanthelasma

## Abstract

**Objective:**

To observe effect of surgery on upper eyelid blepharoplasty in an Asian population.

**Methods:**

This retrospective study was conducted at Armed Forces Institute of Ophthalmology from May 2023 to July 2023. This study included patients from 30 to 70 years of age. Patients with upper double eye lid (dermatochalasis) and xanthelasma were included in this study. Patients were counseled about the risk of surgery. Subcutaneous 0.5% lignocaine, approximately 0.6 ml was given after skin marking in each eye. Distance between eyelid margin and inferior margin eyebrow was marked 21mm, to avoid lagophthalmos. Wound approximation was done with 7-0 vicryl interrupted sutures. Follow up was done at day one and two weeks after upper lid blepharoplasty.

**Results:**

After a follow up of 02 weeks of 35 subjects who underwent upper eyelid blepharoplasty, 24(68.4%) were females while 11(31.4%) were males. The mean average age + SD in years was 53.45± 1.06. All subjects underwent bilateral upper eyelid blepharoplasty in one sitting. Redo surgery was done in one (2.84%) subject after six months. Eyelid oedema was Grade-II on day one postoperatively and was absent after two weeks of follow up in all subjects. However, no lagophthalmos was seen in any of the subject.

**Conclusion:**

Upper Eyelid blepharoplasty is the procedure of choice of correcting double eyelid and xanthelasma. It not only improves periorbital cosmesis but also plays an important part in improving patient vision and contrast.

## INTRODUCTION

Due to increase in quality of life, expectancy and standards of living, aesthetic concerns of majority of people are evolving.^1^ The upper double eyelid prevalence, > 45 years is approximately 16% and is more seen in men.^1^Double eyelid surgery is one the most common aesthetic procedure being performed for correction of xanthelasma and dermatochalasis. Non surgical methods used to correct xanthelasma / dermatochalasis are Nd-YAG laser, intense pulse light therapy, botulinum toxin injections, trichloroacetic acid used topically for xanthelasma. Various different sutural techniques have been discussed in literature. From buried sutures method to incisional approaches, various techniques have been discussed in the field of upper eyelid blepharoplasty.^2^

However due to complexity of upper eyelid, the upper eyelid is the main structure to determine the functional outcome of this form of aesthetic surgery.^3^ The three most important factors for double eyelid surgery are: cosmetic concerns, visual interpretation and functional aspects.^3^From the surgical perspective, upper lid crease is the landmark for dividing upper eyelid into infrabrow and tarsal (approximately 21mm from upper eyelid margin and 10mm from upper lid crease). The lid fold occurs in infrabrow portion of eyelid and is of different sizes, depending on the laxity of skin.^4^,^5^ Mostly the surgeries are performed under local anaesthesia (lignocaine 0.5% with or without adrenaline).

In the literature, the pain threshold during and after upper eyelid blepharoplasty has been measured with visual analogue scale.^6^,^7^ Flexible suspension (suturing of orbicularis oculi with orbital septum) and rigid fixation (suturing of skin to tarsus or aponeurosis) techniques have been used for upper eyelid blepharoplasty.^8^ Post surgical characteristics include, loose tarsal skin, absence of an eyelid fold and deep upper eyelid sulcus.^9^ Continuous sutures causes more oedema as compared to interrupted sutures. Many post-operative surgical complications can be avoided or minimized in delicate surgical hands and with excellent surgical techniques.^10^The aim of our study was to elaborate the clinical outcomes of upper eyelid blepharoplasty and overall improvement of facial cosmesis.

## METHODS

This study was carried out at Armed Forces Institute of Ophthalmology from May 2023 to July 2023. The upper eyelid blepharoplasty done in tertiary care hospital from Jan 2022 to May 2023 were included.

### Ethical Approval:

The study got after approval from hospital ethical review committee (322/ERC/AFIO dated May 16,2023.

### Inclusion Criteria:

- Patients having double eyelid/ dermatochalasis of either gender between 30-70 years of age. Patients having xanthelasma palpebrarum (Grade-I) were included in this study.

### Exclusion Criteria:

- Patients having icthyosis and thyroid eye disease were not included in this study.

After clinical examination of eyelids and grading of xanthelasma (according to classification of xanthelasma), informed consent was obtained from the patient. The skin was marked from upper eye lid skin crease and laterally a wing was created at an angle of 15 degrees to avoid dog ear formation. The distance from upper eyelid margin up to inferior part of superior orbital rim (or inferior part of eyebrow) was measured with scale, which was about 21 mm, while the horizontal lid marking was started from lateral to upper punctum and ending till lateral canthus wing. The incision was given with radiofrequency monoplar cautery needle and skin was excised. Special care was taken in females due to trimming of eyebrows. Skin more than 21mm was excised along. Only in few cases strips of orbicularis were also removed. Interrrupted 7-0 Vicryl sutures were used to close the skin. Pressure bandage with double eye pad was done postoperatively to reduce oedema. Pre and post procedure pictures were captured. Clinical assessment of post procedure upper eyelid oedema and lagophthalmos (House Brackman Grade-I-VI) was noted on day one and two weeks after blepharoplasty. Eyelid oedema was graded with the help of depth (seen visually) and was graded from 0-4:Grade-O: no clinical oedema Grade-I : Slight pitting (2 mm depth) with no visible distortion that rebounds immediately Grade-II: Somewhat deeper pit (4 mm) with no readily detectable distortion that rebounds in fewer than 15 seconds. Grade-III: Noticeably deep pit (6 mm) with the dependent extremity full and swollen that takes up to 30 seconds to rebound.Grade-IV: Very deep pit (8 mm) with the dependent extremity grossly distorted that takes more than 30 seconds to rebound. Patients were advised to keep head end elevated for 24 hours to reduce eyelid oedema and apply ice packs 3-4 times a day for three days. Postoperative systemic antibiotics and anti-inflammatory medicines were advised for three days.

## RESULTS

Out of 35 subjects who underwent upper eyelid blepharoplasty, 24 (68.5%) were females and 11 (31.4%) were males. Age in years was mean±SD 53.45± 1.06. All 35 (100%) subjects presented with Grade-II upper eyelid oedema on 1^st^ post-operative day, while there was no lagophthalmos noted in any of the operated subjects (Table-I). All subjects underwent follow up after two weeks again and they presented with complete resolution of oedema (Table-I). A redo surgery was done on one (2.85%) subject after a period of 06 months. No major complication was noticed postoperatively among all subjects.

**Table-I:** Outcomes of Upper Eyelid Blepharoplasty (n= 35).

Variables	Frequency
Age (Mean ± SD) in years	53.45 ± 1.06
*Gender*	
Male	11 (31.4%)
Female	24 (68.5%)
*Oedema*	
(1^st^ POD)	Grade-II (100%)
(2^nd^ weeks)	Grade 0 (100%)
Lagophthalmos	Grade-I (100%)
Redo Surgery	01 (2.84%)

## DISCUSSION

Blepharoplasty is one the most powerful facial rejuvenation procedure in aesthetic medicine. Thyroid related eye disease and renal disorders can give eyelid malposition and should be investigated thoroughly before proceeding for any lid surgery.^11^,^12^ Various different methods have been used to give a very fine skin incison such as No.15 barde parker blade, radiofrequency needle and CO_2_ laser are used.^13^ We used radiofrequency monopolar cautery 16 W Cut and 18W coagulation (ERBE ICC 50) in our study. Multiple complications have been reported after upper eyelid blepharoplasty surgery, like oedema, ecchymosis, under correction, overcorrection, lagophthalmos, and a more severe among all is blindness. Keloid formation is a rare complication of eyelid blepharoplasty. An Ocular massage is being advised in cases of over correction along with or without subcutaneous corticosteroid injections or 5-fluorouracil injections can also be given to reduce skin contractures and fibrosis.^14^ We noticed oedema and lagophthalmos in our study. No lagophthalmos, and overcorrection was seen in any of our cases although in one case under correction was noticed which was re-operated after six months. In the literature various indications have been mentioned for upper eyelid blepharoplasty, among which dermatochalasis and xanthelasma are common.^15^ We also included patients with dermatochalasis and xanthelasma in our study.

**Fig.1:**
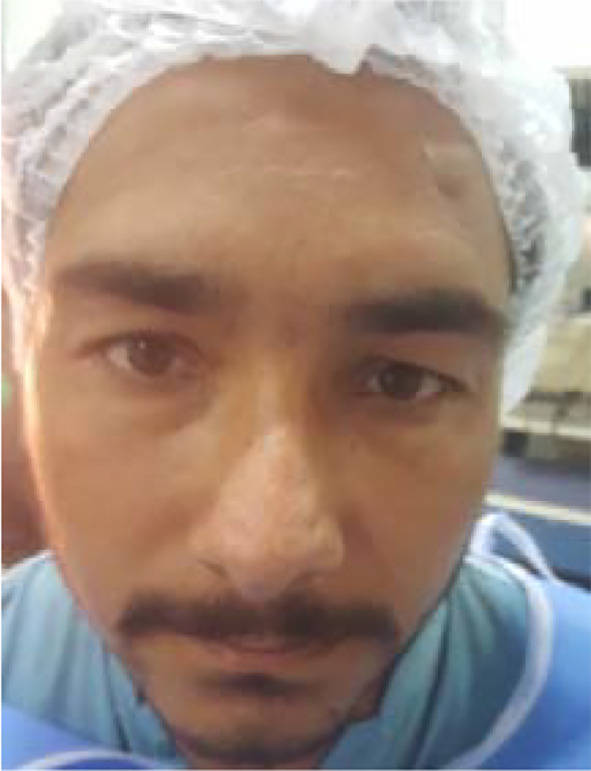
Bilateral Upper eyelid Dermatochalasis.

A tripier flap was used in one of our patient due to excessive xanthelasma involving both upper and lower eyelid. Upper eyelid blepharoplasty provide various different functional and cosmetical outcomes along with improvement of vision, visual fields and headaches.^16^ We also noticed significant cosmetic, functional improvement in our study. The frequency of muscle contraction of frontalis and orbicularis was noticed with the help of electromyography(EMG) before and after upper eyelid blepharoplasty by Maria H.J. Hollander et al, and they observed the reduced frequency and fatigueness of frontalis muscle after upper eyelid blepharoplasty.^17^ In the literature, it is noticed that interrupted suture technique gives better approximation of the tissues and overall patient satisfaction.^18^-^21^ This retrospective study not only provided the results of patient satisfaction but also helped in analysis of clinical outcomes of upper eyelid blepharoplasty.

**Fig.2:**
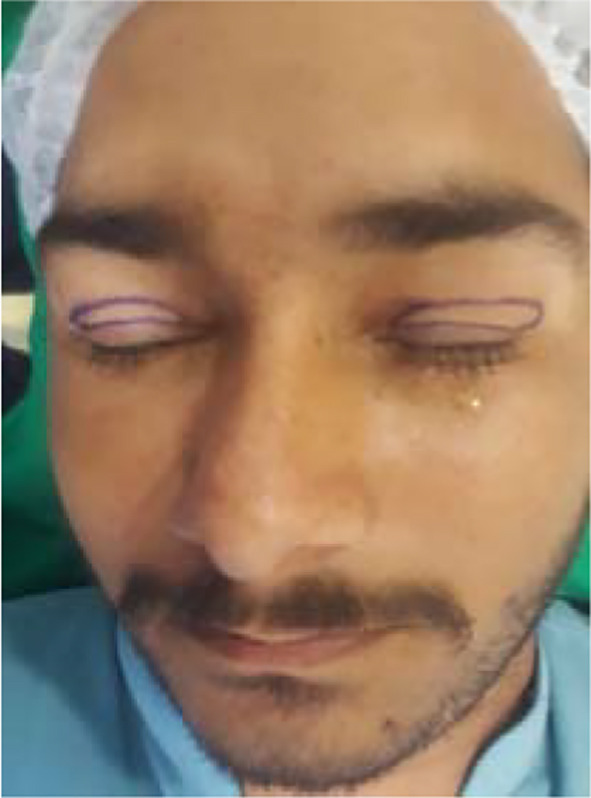
Marking with surgical marker.

**Fig.3:**
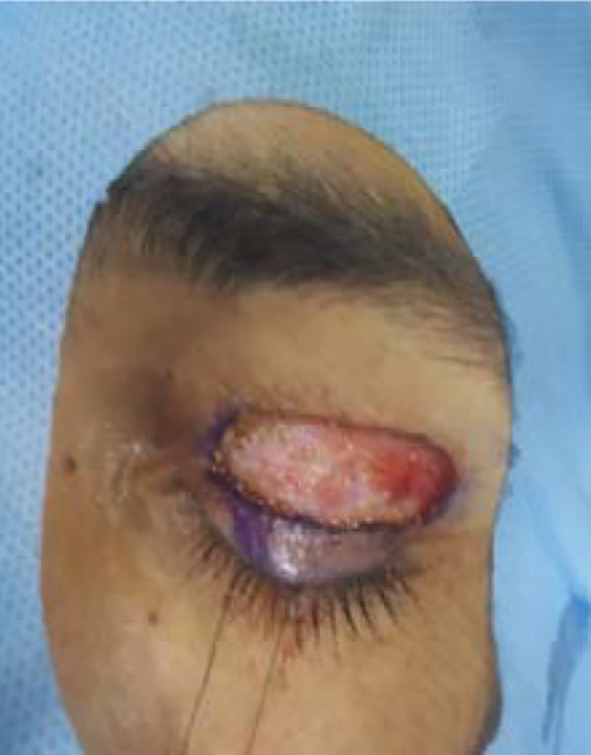
Intraoperative removal of Skin.

### Limitations:

We did not include patients with lower eyelid blepharoplasty. We also did not include patients in which fat pads were removed.

**Fig.4:**
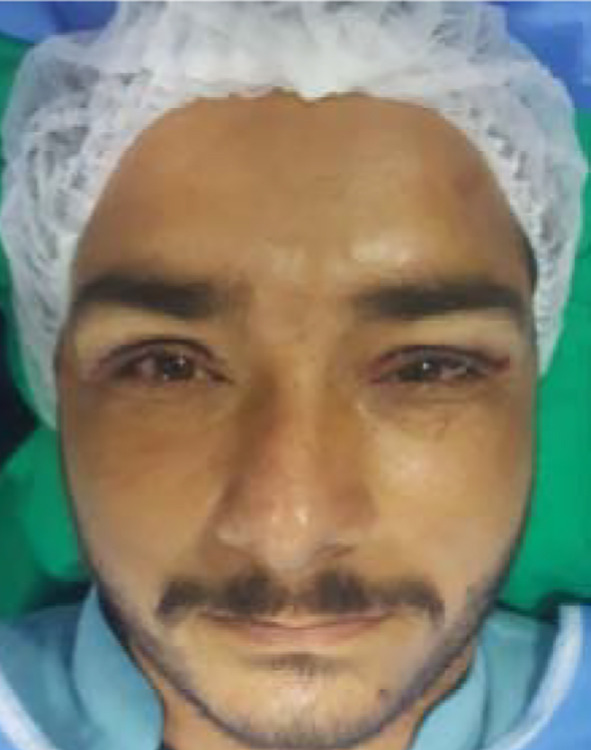
Postoperative after 7-0 vicryl closure.

## CONCLUSION

The upper eyelid blepharoplasty is most common aesthetic procedure done these days. Patient expectations and cosmesis concern should be discussed thoroughly. Post procedure risk factors should be discussed. Blepharoplasty is most successful procedure for treating double eyelid and xanthelasma. It is safe and effective procedure and patient satisfaction rate is also high. Proper skin markings and surgical technique can give better surgical results.

### Author’s contribution:

**MS and R:** Conceived, designed and did statistical analysis & preparation of manuscript, are responsible for integrity of research and approved the final version of the manuscript..
